# Feasibility Pilot of a Randomized Faith-Based Intervention to Reduce Secondhand Smoke Exposure Among Korean Americans

**DOI:** 10.5888/pcd14.160549

**Published:** 2017-02-23

**Authors:** Suzanne C. Hughes, Isabel Corcos, Melbourne Hovell, C. Richard Hofstetter

**Affiliations:** 1 Center for Behavioral Epidemiology and Community Health and Graduate School of Public Health, San Diego State University, San Diego, California

## Abstract

**Introduction:**

Interventions are needed to prevent exposure to secondhand smoke (SHS), which persists in certain immigrant enclaves, including Koreans in the United States. A faith-based and culturally acceptable intervention was developed and pilot tested in collaboration with Korean churches to address SHS exposure among people of Korean descent.

**Methods:**

A pilot cluster randomized intervention trial was conducted with 11 Korean churches in southern California and 75 Korean adults who were exposed to SHS. Study participants received a multicomponent intervention, which consisted of motivational interviewing by telephone and educational materials tailored with related biblical messages; the intervention was bolstered by church-based group activities and environmental cues. The control group received the same type and frequency of intervention components, but the components related only to fruit and vegetable consumption. Data were collected on the feasibility of the intervention and study procedures. SHS exposure and awareness and knowledge of SHS exposure were assessed by telephone interviews at baseline and follow-up.

**Results:**

At follow-up, a larger percentage of the intervention group than the control group reported correct SHS knowledge and disapproval of SHS. The intervention group’s SHS exposure was reduced by 8.5 cigarettes per week (vs a reduction of 1 cigarette per week among the control group).

**Conclusions:**

Initial findings are promising for improving knowledge, attitudes, and protective behaviors surrounding SHS exposure. Results suggest that a faith-based intervention for Korean Americans who are exposed to SHS is feasible, acceptable, and potentially effective in reducing their exposure to SHS.

## Introduction

Secondhand smoke (SHS) exposure is a preventable health hazard. The health effects include heart disease, stroke, lung cancer, asthma exacerbation, other respiratory diseases, and ear infection (1–3). Even brief exposure poses a risk to nonsmokers’ health (1).

Disparities in SHS exposure have been documented for Korean residents in the United States (Korean Americans) and stem from high levels of smoking among men, many of whom emigrated from South Korea when men’s smoking was the norm; for example, in 1998 66% of South Korean men smoked, and in 2010 48% smoked (4). In California, a third of Korean American nonsmokers reported exposure to SHS on a typical day in 2001 and 2002 (5); more than two-thirds of nonsmokers reported having family members who smoked, and even more had friends and associates who smoked. Results from focus groups of Korean Americans indicate that SHS exposure bothered nonsmokers physically and emotionally (6), but avoidance was hindered by cultural norms such as avoiding conflict and respect for elders and male authority.

This study pilot tested an intervention to address the need for culturally acceptable SHS interventions for Korean Americans. Reaching Korean Americans can be challenging because of their lack of access to mainstream health care and limited English proficiency; in 2011, 55% of Korean Americans who spoke Korean at home did not speak English very well (7).

Korean churches are potential forums for health promotion for Korean Americans. Most Korean Americans (70%) attend Korean church (8) and prefer church settings or social networks for receiving health information (9–11). Among the few health intervention studies involving Korean churches, most engaged in community-based participatory research and tested multifaceted screening programs (eg, mammography screening plus education, colorectal cancer screening education plus navigation assistance) (12,13). We describe the development and pilot testing of a church-based intervention for SHS exposure among Korean Americans.

## Methods

### Study design and recruitment

This pilot study assessed feasibility for a cluster randomized design. Churches were randomized to the intervention or control condition. Study participants formed a cluster within each church. The pilot was approved by the institutional review board at San Diego State University. The study was conducted in San Diego County from 2009 to 2012.

From mid-2009 to mid-2010, the study team met with leaders of the 11 largest Korean churches (40 to ≥900 members; 7 Presbyterian, 2 Baptist, 1 Catholic, and 1 nondenominational) on several occasions to discuss collaboration. They all agreed to participate in the study. We combined the 2 smallest Presbyterian churches and matched them and the other 9 churches on denomination similarity before randomization to treatment group.

In late 2010, study staff offered a voluntary, 2-page screening questionnaire in English and Korean to adults after a church service and announcement by the pastor, study church coordinator, or church team. The questionnaire requested a respondent’s contact information and signature permitting contact by the study. Of 1,093 screening respondents, 253 met the following inclusion criteria: aged 18 years or older, of Korean descent, current nonsmoker, and SHS exposure of at least 1 cigarette per week. An additional criterion of not planning to move from the county in the next 6 months, which was determined at a baseline telephone interview, was applied before enrollment into the study. Research staff attempted telephone contact of eligible respondents sequentially by screening identification number within each church until we met our goal of at least 3 participants per church cluster and 30 participants per treatment group after attrition. Of 79 eligible screening respondents invited to participate in the trial, 2 refused (1 per group), and 77 enrolled. After 1 dropout per group (due to hectic schedules), 75 participants (35 in the control group, 40 in the intervention group) completed the study. The number of enrolled participants per church cluster ranged from 4 to 11. Researchers obtained verbal informed consent before the baseline interview and mailed a copy of the consent form to participants.

### Intervention

**Intervention development.** We used a community-based participatory research approach (14,15). During 2010, study staff and church teams (composed of 2 to 3 well-known and respected members from each church) collaborated on the intervention development. The study and church teams met several times to discuss the content and delivery of the different components of the intervention. They discussed findings from our research with Korean Americans (5,6,16,17), such as the sources of SHS exposure (mostly male smokers), nonsmokers’ concealed dislike of SHS, and nonsmokers’ lack of skills to circumvent exposure under the constraints of cultural norms, such as avoiding conflict to maintain harmony and respecting male or elder authority in the family. Before recruitment of churches began, researchers completed 15 in-person key informant interviews with leaders from the Korean church, radio, business, student, and health communities. The informants highlighted smoking as a major issue for their community, smokers’ difficulty in quitting, lack of access to Western medicine, and Koreans’ tendency to seek help or advice from their church, family, or Korean friends. To increase salience and acceptability, the intervention was designed for compatibility with Korean norms (eg, elder respect, group-mindedness), informed by previous research findings and input by church teams. The multilevel intervention incorporated individual and sociocultural factors, as recommended by the Behavioral Ecological Model (18,19). The multicomponent intervention was also loosely modeled after African American church-based diet interventions (20) and consisted of coaching, materials, and church activities, in both English and Korean. The intervention components were designed to be complementary in tackling the different contingencies and social dynamics of SHS exposure.

**Coaching.** A bilingual (English/Korean) staff conducted 5 (approximately weekly) motivational interviewing sessions with enrolled individual participants by telephone. Coaches were trained in motivational interviewing techniques (21) (eg, open-ended questions, affirmation, reflective listening) with specific examples on SHS. Sessions averaged approximately 20 minutes. The coach tailored the session to the participant’s situation and readiness level to reduce SHS exposure. The coach-guided goal-setting and strategies (eg, avoidance, environmental cues [displaying no-smoking signs and removing ashtrays]), which were reviewed at subsequent sessions.

**Materials.** Enrolled participants received a binder containing information about SHS constituents and health-related effects and exercises to identify and align their values with reducing SHS exposure. The binder incorporated biblical scriptures with health themes. The binder also described strategies to reduce SHS in specific locations, including how to create a smoke-free home; how to avoid exposure in cars, at school, at work, in public spaces, and in other social settings; and how to encourage and support any smokers in their networks to quit smoking.

Participants received weekly diary worksheets (to document goals, strategies, and progress), study logo items (refrigerator magnet, magnetized pen, and reusable grocery bag), and coasters and no-smoking signs to display in locations of SHS exposure.

**Church activities.** Study participants were encouraged by their coaches to attend an SHS informational group presentation and discussion led by the study church coordinator and a church team leader at their church. Most of the activities occurred in late 2010 and early 2011, after church services or during small group meetings and were announced in the church bulletin or at the church service. Church activities were open to the entire congregation to promote community awareness and support and to alter the social norms regarding SHS. The study church coordinator and a church team member introduced the study and led a PowerPoint (Microsoft Corp) or handout presentation and discussion of the constituents and health effects of SHS, why Koreans experience higher SHS exposure than other groups, and how to avoid SHS exposure in their daily lives. Church activities lasted 1 to 1.5 hours. Attendees were offered SHS brochures, quit-smoking guides, SHS stickers, reusable grocery bags, and insulated lunch bags with the SHS study logo as a reminder to protect themselves and to promote the educational theme in public, particularly outside Korean markets and businesses where smoking occurred.

### Control condition

To increase participation and comparability between treatment conditions, control group participants received a fruit and vegetable (F&V) intervention with similar components: 5 motivational interviewing coaching sessions, materials (binder, diary worksheets, magnet, pen, and bag), and church activities. The development process and timeline for the F&V intervention was the same as described for the SHS intervention, but the content and logo pertained only to increasing F&V intake.

### Interviews

Trained bilingual research staff (other than coaches) conducted baseline and follow-up (within a week after coaching ended) telephone interviews with enrolled participants, in English or Korean. Interviews averaged 45 minutes. Participants received $10 and $30 for baseline and follow-up interviews, respectively. Participants reported demographic and other characteristics (Table 1). Both interviews asked about SHS attitudes, knowledge, and exposure.

**Table 1:** Participants’ Baseline Characteristics, by Treatment Group, Study on Exposure to Secondhand Smoke Among Korean Americans, San Diego County, United States, 2009–2012

Characteristic	Control (n = 35)	Intervention (n = 40)	Total (N = 75)	*P* Value^a^
Female, n (%)	20 (57)	28 (70)	48 (64)	.25
Married, n (%)	18 (51)	26 (65)	44 (59)	.23
Graduated high school, n (%)	33 (94)	39 (98)	72 (96)	.60
Employed, n (%)	20 (57)	16 (40)	36 (48)	.14
Completed interview in Korean, n (%)	34 (97)	38 (95)	72 (96)	.64
Spoke Korean only or mostly, n (%)	18 (51)	24 (60)	42 (56)	.46
Born in Korea, n (%)	32 (91)	35 (88)	67 (89)	.58
Age, mean (SD), y	40 (15)	36 (11)	38 (13)	.19
Age moved to the United States, mean (SD), y	30 (15)	29 (11)	29 (13)	.74
Attend religious services, mean times/mo (SD)	4 (3)	6 (4)	5 (4)	.17

Abbreviation: SD, standard deviation.

a *P* value derived from independent *t* tests for continuous variables and derived from Pearson or Fisher exact χ^2^ tests for categorical variables.

To assess attitudes, participants rated their approval or disapproval (strong, somewhat) for smoking indoors where nonsmokers lived and smoking by men and women among nonsmoking friends. To assess knowledge, participants were asked, “How many substances does cigarette smoke contain: less than 100, 100–1,000, more than 1,000, or don’t know?” They were also asked whether SHS causes heart disease. Weekly SHS exposure was estimated from reported frequency of exposure in the past month (never, 1–3 times in past month, 1 to 2 times/wk, 3 to 4 times/wk, 5 to 6 times/wk, or daily), and the usual number of cigarettes on days exposed. To assess network smoking, participants specified categories of their network (eg, friends, coworkers, family) seen at least monthly. To assess SHS avoidance, participants indicated their response (ignore, hint, ask smoker to stop, or move) to someone smoking nearby. Participants identified who in their network smoked and who smoked in their presence. 

Participants’ feedback was obtained at the follow-up interview. Participants reported whether their pastor’s sermons or congregation members discussed smoking or SHS, whether they attended church activities, and whether activities were helpful. Participants rated their coaching sessions according to how easy it was to make time for sessions, the number of sessions, session length, whether the coach listened, the helpfulness of goal-setting, and the amount they learned. With regard to educational materials, participants reported whether they read their binder and used their pen, magnet, stickers, coaster, signs, or bag. Regarding the perceived effectiveness of the program, participants noted results from participating, their level of confidence to maintain results (very, somewhat, not very), what affected their goals, whether they encouraged anyone to quit smoking, and whether a smoker in their network tried to quit.

### Evaluation

Process data were collected during each stage of the study (eg, contacts with churches, intervention development, screening, participant recruitment and retention, interview completion, coaching calls, and church activities). Feasibility of urine collection was assessed in the last 4 control and 4 intervention enrolled participants for the 7 days preceding baseline and follow-up interviews. Participants received urine collection kits and verbal and written instructions to collect a first morning urine sample on a Saturday, Sunday, or Monday (representing weekend SHS exposure) and another sample from Tuesday through Friday (representing nonweekend exposure). This schedule considered the half-life of cotinine, a metabolite of nicotine and a biomarker of SHS exposure (22). Eight participants were recruited to provide a urine sample, and samples were retrieved from participants during a home visit.

Descriptive statistics for attitudes, knowledge, and SHS-related behaviors were analyzed using SPSS (version 22, IBM Corp). As a feasibility pilot, the study was not powered for significance testing for impact outcomes (23); however, estimates and 95% confidence intervals were calculated. Significance was set at *P* < .05.

## Results

### Feasibility

Collaboration with churches was feasible. Allotting time for ongoing interactions with churches facilitated trust and involvement in the study and helped with changes in church leadership. Feasibility of conducting a faith-based intervention was demonstrated. Screening at churches was efficient; more than 20% of the 1,000 or more completed questionnaires were eligible. Among the eligible subset invited for the pilot, 97% enrolled.

Retention was high (97%) for completing follow-up interviews. All 8 participants who were recruited to provide urine samples provided the requested samples. Treatment fidelity was moderately high; an average of 4.8 sessions were completed, and 85% of participants completed all coaching sessions. Church activities to promote the intervention were well-received. Attendance ranged from 25 people in the smallest church to approximately 300 in the largest church.

### Baseline characteristics

Participants’ mean age was 38 years, 64% were female, 59% were married, 96% completed high school, and 48% were employed (Table 1). Most participants were born in Korea (89%) and completed the interview in Korean (96%). Church attendance averaged 5 times per month. There were no significant differences between treatment groups.

### Changes in attitudes and knowledge

At baseline, strong disapproval was widespread (83% among controls, 78% among the intervention group) for smoking in the home by someone living with nonsmokers (Table 2). At follow-up, strong disapproval grew to 89% among control group participants and to 100% among intervention participants.

**Table 2:** Participants’ Attitudes, Knowledge, and SHS-Related Behaviors, by Treatment Group and Time Point, Study on Exposure to SHS Among Korean Americans, San Diego County, United States, 2009–2012^a^

Item	Control Group (n = 35)			Intervention Group (n = 40)		
	Baseline	Follow-up	∆ (95% CI)	Baseline	Follow-up	∆ (95% CI)
**Questions about attitudes about SHS (answer, “disapprove strongly”)**						
Smoking inside home by those living with nonsmokers	29 (83)	31 (89)	6 (−10 to 21)	31 (78)	40 (100)	23 (10 to 35)
Man smoking near nonsmoker friend	10 (29)	17 (49)	20 (3 to 37)	8 (20)	30 (75)	55 (37 to 73)
Woman smoking near nonsmoker friend	13 (37)	18 (51)	14 (2 to 28)	11 (28)	31 (78)	50 (32 to 68)
**Questions about knowledge of SHS (correct answer given)**						
No. of substances in SHS	12 (34)	19 (54)	20 (5 to 35)	22 (55)	39 (98)	43 (27 to 58)
SHS causes heart disease	29 (83)	30 (86)	3 (−7 to 13)	32 (80)	39 (98)	18 (6 to 29)
**SHS-related behaviors**						
Mean no. of cigarettes/wk of SHS exposure	7.6	8.6	1.0 (−3.1 to 5.1)	9.8	1.3	−8.5 (−12.2 to −4.9)
Response to smoking nearby, move or ask not to smoke	20 (57)	22 (63)	6 (−10 to 21)	27 (68)	35 (88)	20 (6 to 34)
**Smoking in participant’s regular network**						
Spouse smokes in participant’s presence	4 (11)	3 (9)	−3 (−8 to 3)	14 (35)	0	−35 (−50 to −20)
Male friend smokes in participant’s presence	13 (37)	9 (26)	−11 (−27 to 4)	15 (38)	6 (15)	−23 (−37 to −8)
Female friend smokes in participant’s presence	3 (9)	2 (6)	−3 (−8 to 13)	4 (10)	1 (3)	−8 (−16 to 1)
Coworker smokes in participant’s presence	4 (11)	7 (20)	9 (−1 to 18)	5 (13)	0	−13 (−23 to −2)

Abbreviations: CI, confidence interval; SHS, secondhand smoke.

a Some columns do not total 100% due to rounding; ∆ indicates change in % or mean from baseline to follow-up within group, and values are rounded. All values are no. (%), unless otherwise indicated.

At baseline, participants were less likely to report strong disapproval for men’s smoking in the presence of nonsmoking friends (29% among controls, 20% among intervention participants) than for women’s smoking in the presence of nonsmoking friends (37% among controls, 28% among intervention participants). By follow-up, strong disapproval increased for women’s smoking (51% among controls and 78% among intervention) and men’s smoking (49% among controls and 75% among intervention) among nonsmoker friends.

Knowledge of the number of substances in tobacco smoke increased from baseline (34% among controls, 55% among intervention participants) to follow-up (54% among controls; 98% among intervention participants). At baseline and follow-up, knowledge that SHS causes heart disease was high (80% to 98%).

### Changes in SHS exposure

Controls’ exposure to SHS increased by 1.0 cigarettes per week from baseline (7.6 cigarettes/wk) to follow-up (8.6 cigarettes/wk). Intervention participants’ exposure declined 8.5 cigarettes per week (from 9.8 at baseline to 1.3 at follow-up). For SHS avoidance behaviors, moving away or asking the smoker not to smoke increased from baseline (57% of controls; 68% of intervention participants) to follow-up (63% for controls, 88% for intervention participants). At baseline, participants’ spouses, male friends, or coworkers were the most likely networks to smoke in the participant’s presence in both groups (control and intervention). At follow-up, networks’ smoking in the participant’s presence declined, with a larger decline among intervention participants. 

### SHS intervention participants’ assessment

Most intervention participants reported that smoking or SHS was discussed by their pastor in a sermon (73%) and other church members (85%). Nearly all (95%) participants attended church activities. All attendees rated the activities as helpful.

Most participants (75%) found it easy to make time for their coaching sessions. Most (90%) rated the number and length of sessions as “just right.” For most participants (62%), the amount learned was sufficient, but for the rest it was too much. Everyone reported that the coach listened, and 97% found goal setting helpful. All participants in the intervention group read at least some of the binder materials and used the pen. Nearly everyone used the stickers and magnet, 85% used the bag, 72% used coasters, and 62% used the signs.

Regarding the perceived effectiveness of the intervention, participants were “very” confident (95%) or “somewhat” confident (5%) about maintaining results. Most participants (95%) had encouraged someone to quit smoking, 75% said that someone with whom they spent time had attempted to quit, and 10% said that someone had quit. Regarding achieving their goals, three-fourths of participants indicated that coaching helped, 15% mentioned the binder, and others mentioned a spouse’s support or avoidance behaviors such as spending less time with network smokers. Common challenges were talking to a smoker who was a male family member (husband, father), a friend, fellow church member, coworker, boss, or customer. Benefits included learning about SHS and related health risks, increasing awareness of SHS, and skills to encourage smoking cessation and protect themselves.

## Discussion

This pilot study demonstrated feasibility for a faith-based, multilevel intervention for preventing exposure to SHS among Korean Americans. Changes in knowledge, attitudes, and behaviors were in the predicted direction. This pilot reiterates the importance of budgeting time and a study church coordinator to develop, coordinate, and sustain partnerships with churches. One year was needed to recruit churches and form collaboration. Ongoing contacts helped to maintain collaboration despite church leadership turnover in a few churches.

Data collection and urine collection were feasible and culturally appropriate assessment tools for this population. Screening at church sites provided credibility for the study and were efficient channels for recruitment. Using study staff with backgrounds similar to those of participants and study materials in Korean and English addressed participants’ language needs.

SHS exposure is influenced by physiological, environmental, and cultural factors. Previous surveys of Korean Americans show that social reprimand discouraged smoking (16). In this study, the link between physical and spiritual health was embraced by the church leaders and congregants. Involving church peers was important. Church activities were well-attended by study participants and fellow churchgoers and stimulated discussions and social support for not smoking. In addition, most pastors mentioned SHS-related health messages in their sermons as part of their mission.

Intervention effect was achieved across multiple domains — attitudes, knowledge, and exposure (improvement from 18% to 55% for attitudes and knowledge and an 8.5 cigarette/wk reduction in SHS exposure among intervention participants). For comparison, there are few, if any, other intervention research activities that have focused on reducing SHS exposure among adult nonsmokers; most of the previous SHS interventions have targeted children’s exposure, pregnant women (24,25), adult smoking cessation, hospitalized patients, or household smoking bans.

Areas for refinement for this study include participants’ finding time for coaching, reducing the information content or making it more relatable, and increasing assertiveness skills, eg, adding role-playing with multiple strategies and sample “responses” for when a relative or friend smokes near them.

This study had limitations. This pilot study was not designed to test hypotheses for the outcomes measures; the estimates should be interpreted with caution as they may be unreliable or biased due to the small sample and self-reported data. Although adult-reported exposure is used commonly and has shown moderate reliability and validity (1), corroboration with a biomarker such as urine cotinine is recommended for full trials. In this pilot, asking participants about each category of network smoking and smoking in the participant’s presence was used as an aid in recalling the instances and amount of SHS exposure.

Overall, the study results are promising for reducing SHS exposure and warrant further research to test the efficacy of this culturally appropriate approach and intervention.
